# Central-stimulating and analgesic activity of the ethanolic extract of *Alternanthera sessilis* in mice

**DOI:** 10.1186/1472-6882-14-398

**Published:** 2014-10-15

**Authors:** Himangsu Mondal, Sanjib Saha, Khalijah Awang, Hemayet Hossain, Abdulwali Ablat, Md Khirul Islam, Ismet Ara Jahan, Samir K Sadhu, Md Golam Hossain, Jamil A Shilpi, Shaikh J Uddin

**Affiliations:** Phytochemistry and Pharmacology Research Laboratory, Pharmacy Discipline, Khulna University, Khulna, 9208 Bangladesh; Department of Biochemistry, Faculty of Mathematics and Natural Sciences, University of Turku, Turku, FI 20500 Finland; Department of Chemistry, Faculty of Science, University of Malaya, 50603 Kuala Lumpur, Malaysia; BCSIR Laboratories, Bangladesh Council of Scientific and Industrial Research (BCSIR), Dhaka, 1205 Bangladesh; Institute of Biological Science, University of Malaya, 50603 Kuala Lumpur, Malaysia; Centre for Natural Products and Drug Discovery, University of Malaya, 50603 Kuala Lumpur, Malaysia

**Keywords:** *Alternanthera sessilis*, Central stimulating activity, Pentobarbitone induced sleeping time, Acetic acid induced writhing test

## Abstract

**Background:**

*Alternanthera sessilis* is a popular vegetable and used in traditional medicinal practice of Bangladesh and other parts of Asia to relive tiredness, laziness, and sleeps as well as pain and inflammation. However, no report was found on the neuropharmacological and analgesic activity of this plant to-date. Present study was undertaken to evaluate the neuropharmacological and analgesic activity of the ethanol extract of *A. sessilis* whole plant (ETAS) in mice models.

**Methods:**

Central stimulating activity was investigated by pentobarbitone induced sleeping time, open field, and hole cross tests. Analgesic activity was evaluated by acetic acid induced writhing and hot-plate methods. The tests were performed at 250 and 500 mg/kg body weight dose levels.

**Results:**

In sleeping time test, ETAS significantly (p < 0.001) increased the onset of sleep, and decreased the duration of sleep. In open field and hole cross tests, ETAS significantly (p < 0.001) increased the movements of mice which persisted throughout the study period. In writhing test, ETAS showed, significant (p < 0.001) inhibition of writhing reflex. In hot plate test, ETAS significantly (p < 0.001) raised the pain threshold. In HPLC analysis for polyphenols, (+)-catechin, rutin, ellagic acid, and quercetin were detected in ETAS (117.72, 490.74, 3007.26, and 13.85 mg/100 g of dry extract, respectively).

**Conclusion:**

Present study supported the traditional uses of *A. sessilis* and indicated that the plant can be a potential source of bioactive molecules.

## Background

*Alternanthera sessilis* (L.) R.Br. ex DC. (Family: Amaranthaceae) is an aquatic plant commonly growing in Bangladesh. In Bangladesh, it is known as Chanchi, Haicha, Sachi-shak, and distributed all over the country especially in moist areas. It also grows in many other Asian countries including India, Nepal, Sri Lanka, Malaysia, Indonesia, China, and Taiwan [1].

*A. sessilis* is widely used as vegetable in Asia, and occasionally cultivated for its use in herbal medicine. Traditionally, the leaves of *A. sessilis* is used in skin diseases, eye diseases, wound healing and as an antidote for snake bite [2]. In local communities of Bangladesh and other South Asian countries, the plant is used in the treatment of rheumatism and painful swellings associated with wounds. A decoction of *A. sessilis* is also used to alleviate pain and intestinal inflammation [3]. The plant *A. sessilis* is well known for its stimulant activity and used for removing tiredness, laziness, and sleeps [1]. In some parts of India, including Bihar, poultice of pounded fresh material is used for sprains, burns, and eczema [4]. The plant is also used in the treatment of malaria, diarrhoea, dysentery, post-natal complaints, night blindness, and helminthiasis [1]. Anthelmintic activity is known to be produced when the juice of *A. sessilis* is administered with two spoons of warm water in an empty stomach [1].

The plant is reported to have diuretic [5], haematinic [6], antioxidant [7], anti-inflammatory [4], cytotoxic [8], antipyretic [9], hepatoprotective [10], antiulcer [11], antimicrobial, and wound healing [12] properties. The herb is also reported as febrifuge, galactagogue, abortificient, and used in the treatment of indigestion [13]. The plant is reported to contain lupeol, α and β-spinasterol, β-sitosterol, stigmasterol, and campesterol [14, 15].

Although the plant has traditional uses as pain killer or stimulant (removes tiredness, laziness, and sleeps), but there is no scientific report to-date on its neuropharmacological and analgesic activity. As a part of our continuing screening of medicinal plants with analgesic and neuropharmacological activity [16–18], here we now report the central-stimulating and analgesic activity of the ethanol extract of *A. sessilis* in mice models.

## Methods

### Plant material

Whole plants of *A. sessilis* were collected from Gopalgonj, Bangladesh in October 2011, and were identified by the experts at Bangladesh National Herbarium, Dhaka. A voucher specimen (DACB 36542) was deposited there for future reference. Plants were washed to remove extraneous materials and then shed dried for several days. Shed-dried plant material was ground into a coarse powder with the help of a mechanical grinder, and the plant material was kept in air tight container until the extraction process commenced.

### Extraction

Powdered plant material (380 g) was extracted with 90% ethanol in distilled water for three days with occasional shaking. The extract was filtered and dried in a rotary vacuum evaporator at 45°C under reduced pressure to obtain the crude extract (ETAS) (yield: 5.3% extract of dried plant material).

### Test animals

Young Swiss Albino mice of either sex, aged 4–5 weeks and average weight of 20–25 g were purchased from the Animal Resources Branch of International Centre for Diarrhoeal Disease Research, Bangladesh (ICCDR,B) and used for the pharmacological investigations. The animals were kept in polypropylene cages under standard laboratory condition maintained at room temperature of 25 ± 2°C, relative humidity of 56-60%, and proper dark–light cycle. The animals were supplied with ICDDR,B formulated rodent food and water ad libitum*.* The experimental protocols were approved by the ethical committee of Pharmacy Discipline, Life Science School, Khulna University, Bangladesh.

### Chemicals and drugs

Gallic acid, (+)-catechin hydrate, vanillic acid, caffeic acid, (-)-epicatechin, *p*-coumaric acid, rutin hydrate, ellagic acid, and quercetin were obtained from Sigma-Aldrich (St. Louis, MO, USA). Acetonitrile (HPLC), methanol (HPLC), acetic acid (HPLC), and ethanol were purchased from Merck (Darmstadt, Germany). Tween-80 was obtained from Loba Chemie Pvt Ltd, India. Diclofenac sodium, caffeine (Beximco Pharmaceuticals Ltd, Bangladesh), morphine (Popular Pharmaceuticals Ltd, Bangladesh), and pentobarbitone (Incepta Pharmaceuticals Ltd, Bangladesh) were used as reference drugs in the present study.

### Grouping and dosing

Animals were randomly divided into four groups, each comprising six animals. Control group was treated with Tween 80 (1% w/v in water) at a volume of 10 ml/kg; extract groups were treated with ETAS at doses of 250 and 500 mg/kg; positive controls were treated with caffeine (20 mg/kg) for pentobarbitone-induced sleeping time, morphine (5 mg/kg) for hot plates test, or diclofenac sodium (25 mg/kg) for writhing test. Administration of *A. sessil* extract was assumed to be safe based on its traditional use [1, 2, 11] as well as previous studies made using the plant [4, 10, 12]. These reports were also used as a basis for dose selection. Both test and control treatments were administered orally except for hot-plate test. In hot-plate test, intraperitoneal (i.p.) route was selected for administration of the treatments.

### Pentobarbitone-induced sleeping time test

Test groups and control received respective treatments at proper doses as mentioned earlier, while the positive control group received caffeine at the dose of 20 mg/kg, i.p.. Thirty minutes later, pentobarbitone (50 mg/kg, i.p.) was administered to each mouse to induce sleep. Onset of sleep and duration of sleep were recorded for each group and compared with the control [18].

### Open field test

Experimental mice of test groups and control were administered with the respective treatments at the proper doses as mentioned earlier. The mice were placed on the floor of an open field chamber (100 cm × 100 cm × 40 cm) divided into series of squares colored black and white in an alternative format. The number of squares visited by each group was recorded for 3 min at 0, 30, 60, 90, 120, 180, and 240 min during the observation period and compared with the control [18].

### Hole cross test

For the hole cross test, a plastic made transparent cage (30 cm × 20 cm × 14 cm) was provided with a partition fixed in the middle of the cage. A hole of 3 cm in diameter was made in the partition at the height of 7.5 cm above the floor of the plastic cage. Experimental mice were provided with the respective treatments at the proper doses. Each animal was placed in the chamber on either side of the partition, and the number of hole crossed by each group was recorded for 3 min at 0, 30, 60, 90, 120, 180 and 240 min during the observation period. Results of the test groups were compared with the control [17].

### Acetic acid induced writhing test

Analgesic activity of ETAS was evaluated by acetic acid induced writhing method [19]. Test groups and control received respective treatments at the proper doses, while the positive control group received diclofenac sodium (25 mg/kg, p.o.). Thirty minutes later, acetic acid (0.7%, 10 ml/kg, i.p.) was administered to each mouse to induce abdominal contraction known as writhing [20]. After an interval of 5 min, number of writhes for each group was counted for 10 min and recorded. The number of writhes of test groups at different dose levels, and standard were compared with the control.

### Hot-plate test

Mice of both sexes were screened based on their response when subjected to hot-plate. Test groups and control received their respective treatments at the proper doses as mentioned earlier, while the positive control group received morphine (5 mg/kg, i.p.). Pain stimulus was produced by placing the animals on hot-plate maintained at the temperature of 55 ± 0.5°C [21]. Paw licking or jumping off the plate was considered as response to pain stimulus. Reaction time for each group was recorded at 0, 30, 60, 90 and 120 min during the observation period [22]. To avoid any possible accidental paw damage, a cut-off point of 15 sec was considered. Reaction time of the extract and standard was compared with the control [23].

### Phytochemical group test

Phytochemical group test of ETAS was performed using standard test methods for the identification of reducing sugars, alkaloids, steroids, tannins, glycosides, gums/carbohydrates, flavonoids, and saponins [24].

### HPLC analysis for polyphenolic constituents

HPLC analysis was conducted on a Dionex UltiMate 3000 Rapid Separation LC (RSLC) system (Thermo Fisher Scientific Inc., MA, USA) following the method as described by Chuanphongpanich and Phanichphant with some modifications [25]. A quaternary rapid separation pump (LPG-3400RS) and rapid separation photodiode array detector (DAD-3000RS) were coupled with the system. Compounds were separated on an Acclaim® C_18_ (4.6 × 250 mm; 5 μm) column (Dionex, USA) at 30°C using a temperature-controlled column compartment (TCC-3000). Data acquisition, peak integration, and calibrations were accomplished using Dionex Chromeleon software (Version 6.80 RS 10). A gradient elution was programmed with 5%A/95%B from 0 to 9 min, 10%A/80%B/10%C from 10 to 19 min, 20%A/60%B/20%C from 20 to 30 min, flushing and post run equilibration of the column with 100%A for 5 min where A, B and C stand for acetonitrile, acetic acid solution of pH 3.0 and methanol, respectively. The flow rate was kept constant throughout the analysis at 1 ml/min and the injection volume was 20 μl. For detection of the compounds; the UV detector was adjusted at 280, 320, and 380 nm consecutively for 18, 24 and 30 min while the photodiode array detector was adjusted to acquisition range from 200 to 700 nm throughout the entire run time. For calibration curve preparation, a stock solution was prepared in methanol containing ellagic acid, gallic acid, vanillic acid, (+)-catechin, (-)-epicatechin, *p*-coumaric acid, rutin (20 μg/ml each), caffeic acid (8 μg/ml) and quercetin (6 μg/ml). A solution of ETAS was prepared in methanol having the concentration of 5 mg/ml. All solutions (mixed standards, ETAS, and spiked solution) were filtered through 0.2 μm nylon syringe filter (Sartorius, Germany) and degassed in an ultrasonic bath (Hwashin, Korea) for 15 min before HPLC analysis.

### Statistical analysis

Results were expressed as mean ± SEM (Standard error for mean). All statistical analysis was carried out using one-way or two-way analysis of ANOVA followed by Bonferroni’s test. Analysis was performed in Prism 5.0 (GraphPad software Inc., San Diego, CA). Results were considered as significant when p < 0.01.

## Results

### Pentobarbitone-induced sleeping time test

ETAS significantly (p < 0.001) increased the onset of pentobarbitone-induced sleep and decreased total sleeping time in mice at both dose levels. ETAS showed 188.70 and 377.49% increase in the onset of sleep and 12.70 and 23.08% decrease of the duration of sleep at the doses of 250 and 500 mg/kg, respectively (Table 1). Caffeine, used as standard in this study, showed 421.62% increase in the onset of sleep and 38.41% decrease of the duration of sleep at the dose of 20 mg/kg.

**Table 1:** **Effect of ETAS on pentobarbitone-induced sleeping time in mice**

Treatment (***n***=6)	Dose (mg/kg)	Onset of sleep (min)	% Increase of onset of sleep	Duration of sleep (min)	% Decrease of duration of sleep
Control	-	11.24 ± 1.80	-	101.57 ± 2.67	-
Caffeine	20	58.63 ± 1.84*	421.62	62.56 ± 2.23*	38.41
*A. sessilis*	250	32.45 ± 2.89*^,#^	188.70	88.67 ± 2.90^#^	12.70
	500	53.67 ± 2.60*	377.49	78.13 ± 2.31*^,#^	23.08

Values are mean ± SEM. *p < 0.001 vs. control, ^#^p < 0.01 vs. caffeine, the data was analyzed by one way ANOVA followed by Bonferroni’s test.

### Open field test

**Figure 1:**
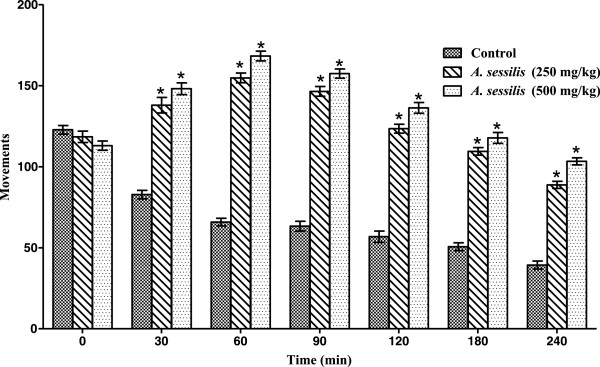
**Effect of ETAS in open field test in mice.** *p < 0.001 vs. control, the data was analyzed by two way ANOVA followed by Bonferroni’s test, values are mean ± SEM (*n* =6).

In open field test, ETAS showed significant (p < 0.001) increase in locomotion in mice from the second observation period (30 min) and continued throughout the entire observation period of 240 min. Maximum effect was noted at 60 min and a successive decrease in stimulating activity was observed with the passage of time for both dose groups (Figure 1).

### Hole cross test

**Figure 2:**
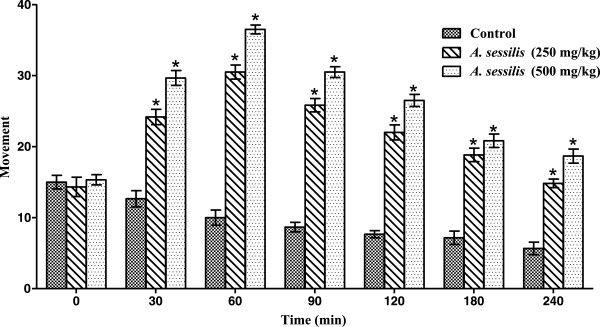
**Effect of ETAS in hole cross test in mice.** *p < 0.001 vs. control, the data was analyzed by two way ANOVA followed by Bonferroni’s test, values are mean ± SEM (*n* =6).

In hole cross test, ETAS significantly (p < 0.001) increased locomotor activity in mice at both dose levels tested as compared to the control. The maximum locomotor activity was revealed at 60 min for both the doses tested, and activity persisted throughout the observation period following a trend of gradual decrease in activity with the passage of time (Figure 2).

### Acetic acid induced writhing test

ETAS showed 37.28 and 59.52% inhibition of writhing at the doses of 250 and 500 mg/kg body weight, respectively, whereas diclofenac sodium showed 76.19% inhibition at the dose of 25 mg/kg as compared to the control, and the results were statistically significant (p < 0.001) (Table 2).

**Table 2:** **Effect of ETAS on acetic acid induced writhing in mice**

Treatment (***n***=6)	Dose (mg/kg)	No. of writhes	% inhibition
Control	-	21.00 ± 0.57	-
Diclofenac sodium	25	5.00 ± 0.36*	76.19
*A. sessilis*	250	13.17 ± 0.47*^,#^	37.28
	500	8.50 ± 0.42*^,#^	59.52

Values are mean ± SEM. *p < 0.001 vs. control, ^#^p < 0.001 vs. diclofenac sodium, the data was analyzed by one way ANOVA followed by Bonferroni’s test.

### Hot-plate test

The ETAS showed maximum reaction time of 6.87 and 7.28 sec at 60 min at the doses of 250 and 500 mg/kg, respectively while morphine showed the maximum reaction time of 10.05 sec at 90 min at the dose of 5 mg/kg (Table 3). The results indicated that the extract significantly (p < 0.001) raised pain threshold as compared to control and the activity was persistent throughout the entire observation period of 120 min.

**Table 3:** **Effect of ETAS in hot-plate test in mice**

Treatment (***n***=6)	Dose (mg/kg)	Reaction time (sec)				
		0 min	30 min	60 min	90 min	120 min
Control	-	4.23 ± 0.07	4.21 ± 0.05	4.18 ± 0.04	4.23 ± 0.05	4.17 ± 0.05
Morphine	5	4.02 ± 0.05	6.03 ± 0.05*	8.03 ± 0.07*	10.05 ± 0.09*	8.91 ± 0.09*
*A. sessilis*	250	4.49 ± 0.06	5.20 ± 0.05*^,#^	6.87 ± 0.03*^,#^	5.92 ± 0.07*^,#^	5.06 ± 0.05*^,#^
	500	4.48 ± 0.07	5.78 ± 0.03*	7.28 ± 0.08*^,#^	6.14 ± 0.05*^,#^	5.51 ± 0.05*^,#^

Values are mean ± SEM. *p < 0.001 vs. control, ^#^p < 0.001 vs. morphine, the data was analyzed by two way ANOVA followed by Bonferroni’s test.

### Phytochemical group test

The phytochemical group test indicated the presence of reducing sugars, alkaloids, steroids, terpenoids, tannins and flavonoids (Table 4).

**Table 4:** **Results of phytochemical group test of ETAS**

Test for phytochemical group	Reagent	Results ^*^
Reducing sugar	Fehling’s test	+
	Benedict’s test	+
Alkaloid	Dragendorff’s test	+
Steroid and terpenoid	Salkowski’s test	+
	Libermann-Burchard reagent	+
Tannin	Ferric chloride test	+
Glycoside	Keller Killiani test (cardiac glycoside)	-
	Borntrager’s test (Anthraquinone glycosides)	-
Gum/Carbohydrate	Molish’s test	-
Flavonoid	Shinoda test	+
	Alkaline reagent test	+
Saponin	Frothing test	-

* “**+**” indicates presence and “-” indicates absence.

### HPLC analysis

**Figure 3:**
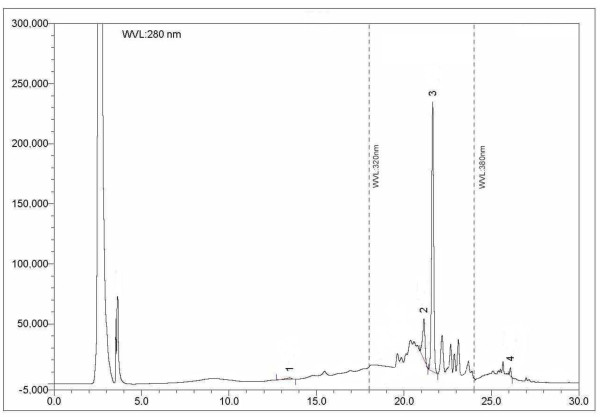
**HPLC chromatogram of ETAS.** Peaks 1: (+)-catechin, 2: rutin, 3: ellagic acid, 4: quercetin.

**Figure 4:**
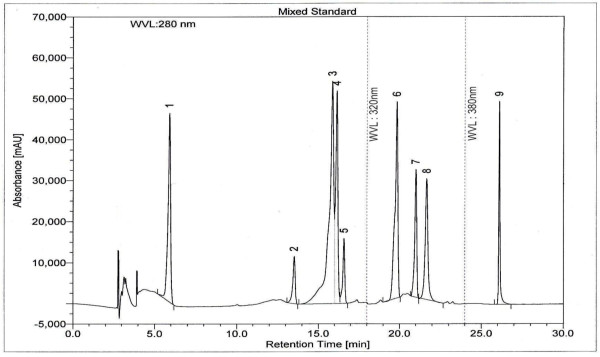
**HPLC chromatogram of a standard mixture of polyphenolic compounds.** Peaks: 1: gallic acid; 2: (+)-catechin; 3: vanillic acid; 4: caffeic acid; 5: (-)-epicatechin; 6: p-coumaric acid; 7: rutin; 8, ellagic acid; 9: quercetin.

HPLC analysis of ETAS showed very high levels of ellagic acid and rutin (3007.26 and 490.74 mg/100 g of extract, repectively). (+)-Catechin and quercetin were also identified but with comparatively lower concentrations (117.72 and 13.85 mg/100 g of extract, respectively) (Table 5). The chromatogram also displayed peaks in regions that represent simple polyphenols, catechins, anthocyanins, flavonoid aglycones and flavonoid glycosides (Figure 3) [26]. Chromatogram of mixed standards is mentioned in Figure 4.

**Table 5:** **Polyphenolic compounds in ETAS identified by HPLC analysis**

Polyphenolic compound	Content (mg/100 g extract)	% RSD
(+)-Catechin	117.72	1.04
Rutin	490.74	1.91
EA	3007.26	3.89
Quercetin	13.85	0.63

RSD: Relative standard deviation.

## Discussion

Pentobarbitone-induced sleeping time, open field, and hole cross tests are most widely used methods for central behavioral analysis and are sensitive ways to evaluate central stimulating activities of drugs and crude plant extracts [27, 28]. Pentobarbitone, a short-acting barbiturate, when given in appropriate dose, induces sedation in animals by stimulating the inhibitory neurotransmitter gamma-aminobutyric acid (GABA) mediated postsynaptic inhibition through allosteric modification of GABA_A_ receptors [29]. In the present study, ETAS significantly increased the time required for the onset of sleep and decreased the duration of sleep as compared to control, which justifies that ETAS might have central stimulating activity. ETAS also increased the locomotor activity in open field and hole cross tests, which further strengthened central stimulating activity of the extract [30]. The probable mechanism of central stimulant effect of the ETAS may be due to the antagonism of inhibitory neurotransmitter, alteration of presynaptic control of inhibitory neurotransmitter release or enhancement of the excitatory neurotransmitter. Quercetin, identified in ETAS, has been reported to have central stimulant effect via antagonism of adenosine A1 receptors [31]. Based on a comparative molecular field analysis model, some flavonoid derivatives were found to be potential adenosine receptor antagonists [32]. The ETAS extract showed positive results for alkaloids and it is well-known that many alkaloids including caffeine, cocaine, cathinone, nicotine, and yohimbine, posess central stimulant effect [33]. Therefore, the identified polyphenols and alkaloids might contribute in the observed central stimulant effect of *A. sessilis* extract.

Several analgesic drugs, including NSAIDs, opiates, and steroids are available for the management of pain, but these drugs are often associated with severe side effects. NSAIDs cause gastric ulceration, opiates can develop dependence, and steroids are associated with side effects affecting hormonal regulation [34]. Many peripherally or centrally acting analgesics have been isolated from plants and thus require extensive studies to explore more analgesic agents from natural sources [35]. Results of the present study revealed that ETAS has peripheral and centrally acting analgesic activity. The plant is reported to contain lupeol [14, 15] and in a recent study, lupeol showed analgesic activity in an inflammatory model of pain through the inhibition of IL-4, IL-5 and IL-13 [36]. The plant is also rich in sterols [14, 15] and therefore, the role of other sterols in the observed analgesic activity cannot be ruled out [37]. In acetic acid induced writhing model, acetic acid increases the level of prostanoids, particularly, PGE_2_ and PGF_2a_ [38] as well as lipoxygenase derived icosanoids in the peritoneal fluid [39]. Abdominal contraction or writhing in mice occurs because of the release of these pain mediators. The extract significantly reduced the number of writhes, and the probable mechanism might involve the inhibition of the release of pain mediators by acting on visceral receptors sensitive to acetic acid.

Hot-plate test is a widely used model for neurologic pain, and centrally acting analgesic agents can increase reaction time in hot-plate test through their action at the spinal cord level [40, 41]. Morphine used as the standard in this study, acts through binding with opioid receptors (μ, δ and κ) present in presynaptic and postsynaptic membrane. The result of hot plate test indicates that the extract also possesses the ability to reduce centrally mediated pain.

In the phytochemical group tests of ETAS, some major phytochemicals, namely, reducing sugars, alkaloids, steroids, terpenoids, tannins, and flavonoids were identified. It is well established that various terpenoids, favonoids, steroids are involved in analgesic activity [42]. In addition, polyphenolic constituents, (+)-catechin, ellagic acid, and rutin identified in the HPLC analysis, which showed analgesic activity in previous studies [43–45]. Phytochemical analysis identified polyphenols, alkaloids and other bioactive compounds in the extract, which can also contribute towards the observed central stimulating and analgesic activities.

## Conclusions

Present study revealed the central stimulating and analgesic activities of ETAS, which strongly supports its use in traditional medicine. Our current research findings demonstrated scientific rationale for the traditional uses of this plant as a central stimulant and analgesic. Interestingly, the extract showed both peripheral and central analgesic activity in established in vivo models. In addition, HPLC and phytochemical analysis identified different bioactive phytoconstituents including polyphenols and alkaloids which might be responsible for observed pharmacological activities. However, further study need to carry out to isolate pure bioactive compounds responsible for these activities with its mechanism of action.
